# Lactate Metabolism: The String-Puller for the Development of Pancreatic Cancer

**DOI:** 10.3390/biology14091213

**Published:** 2025-09-08

**Authors:** Lan Yang, Dong Guo, Kangli Wu, Yiqi Li, Yue Xi, Wenying Qin, Xingzhen Chen, Cefan Zhou, Jingfeng Tang

**Affiliations:** 1 National ‘‘111” Center for Cellular Regulation and Molecular Pharmaceutics, Institute of Biomedical Research, School of Life and Health Sciences, Hubei University of Technology, Wuhan 430068, China; 2 Membrane Protein Disease Research Group, Department of Physiology, Faculty of Medicine and Dentistry, University of Alberta, Edmonton, AB T6G2R3, Canada

**Keywords:** lactate metabolism, lactylation, pancreatic cancer, diagnosis and treatment

## Abstract

Since the discovery of the ‘Warburg effect’ in cancer, lactate is no longer considered merely a metabolic byproduct. It serves as both a metabolic fuel involved in the energy cycle and a signaling molecule that modulates cellular signal transduction. Recent studies have demonstrated that lactate participates in protein lactylation, regulates energy metabolism, reshapes the tumor microenvironment, and facilitates the metastasis of pancreatic cancer. Therefore, targeting lactate metabolism has emerged as a promising strategy to improve therapeutic efficacy and survival rates in pancreatic cancer. In this review, we outline aberrant lactate metabolism and recent advancements in lactylation, and elucidate the biological functions of lactate metabolism in pancreatic cancer, focusing on metabolic reprogramming, angiogenesis, and immune evasion. Additionally, we discuss diagnostic and therapeutic approaches targeting lactate metabolism in pancreatic cancer. Research in this field is critical for understanding the mechanisms driving pancreatic cancer progression and is anticipated to develop novel therapeutic strategies for clinical practice.

## Simple Summary

Pancreatic cancer is a highly aggressive disease with limited treatment options. This review explores a new role for lactate, a molecule once thought to be merely a waste product of pancreatic cancer metabolism. We describe how lactate acts as a key signaling molecule that fuels tumor growth, helps the cancer hide from the immune system, and promotes the formation of new blood vessels. Understanding this process opens up new possibilities for diagnosing and treating pancreatic cancer, potentially by targeting lactate production or its effects within the tumor. This research highlights a promising new direction in the fight against this challenging disease.

## 1. Introduction

Pancreatic cancer, commonly referred to as pancreatic ductal adenocarcinoma (PDAC), is a malignant tumor that primarily originates from ductal epithelial cells. It has long posed a significant challenge in the medical field due to its inconspicuous early symptoms, difficulty in diagnosis, and poor prognosis [1,2,3,4]. Moreover, pancreatic cancer is projected to become the second-leading cause of cancer-related mortality by 2030, second only to lung cancer [5,6,7]. A hallmark of pancreatic cancer is the uncontrolled growth of tumor cells driven by genetic mutations and abnormal activation. These alterations disrupt the normal cell cycle, leading to continuous cancer cell proliferation and tumor formation [8,9], thereby creating a tumor microenvironment characterized by hypoxia and nutrient deficiency [10,11,12]. To survive in such a harsh environment, pancreatic cancer cells reprogram their metabolic pathways to support energy demands, biosynthesis, and redox balance. For example, they rewire glycolysis and glutamine metabolism pathways, generating sufficient adenosine triphosphate (ATP) and biosynthetic intermediates to sustain energy supply [13,14,15,16].

Lactate metabolism, which encompasses the generation, transport, and utilization of lactate, is closely associated with glycolysis and glutaminolysis [17,18]. It plays a crucial role in every stage of pancreatic cancer progression, including tumor initiation, development and metastasis. During tumor initiation, pancreatic cancer cells primarily generate energy through a high rate of glycolysis in the cytosol, even in the presence of oxygen [19,20]. This phenomenon, first proposed by Otto Warburg in 1924 [21,22,23], is known as the ‘Warburg effect’, which leads to abundant lactate production. Lactate not only supplies energy for tumor cells but also provides raw materials for biosynthesis, thereby supporting rapid cancer cell proliferation and tumor growth. As pancreatic cancer progresses and the tumor size increases, the severity of intra-tumoral hypoxia and nutritional deficiencies escalates [7,24]. This drives cancer cells to further reprogram their metabolic pathways, particularly lactate metabolism. Specifically, cancer cells augment lactate uptake by upregulating the expression of related proteins, like monocarboxylate transporters (MCTs) [25,26]. Lactate suppresses prolyl hydroxylase (PHD) activity, preventing the degradation of hypoxia-inducible factor 1α (HIF-1α) under normoxic conditions and enabling its accumulation and translocation to the nucleus [27]. Subsequently, HIF-1α activates the transcription of genes that drive tumor angiogenesis, cell proliferation and metastasis. HIF-1α also promotes glycolysis and the expression of MCTs, thus amplifying lactate production and uptake to create a self-perpetuating cycle. This allows pancreatic cancer cells to acquire additional nutrients by fostering angiogenesis within a hostile microenvironment, while enhancing their migration and invasion abilities [28,29]. In the advanced stages of pancreatic cancer, the extensive metastasis of tumor cells occurs [7,30]. Lactate secreted by cancer cells acidifies the tumor microenvironment by reducing its pH [31,32], which inhibits immune cells, and activating key proteases to degrade the extracellular matrix (ECM), promoting tumor invasion and metastasis [33,34].

In this review, we highlight abnormal lactate levels in pancreatic cancer and focus on lactylation. Given the distinct features of different stages in pancreatic cancer progression, our analysis centers on the impact of lactate metabolism on multiple physiological processes, including metabolic reprogramming, angiogenesis, and immune evasion. Furthermore, we discuss the applications and future challenges of diagnostic and therapeutic approaches targeting lactate metabolism in pancreatic cancer. This review illuminates the role of lactate metabolism in pancreatic cancer, enriching fundamental knowledge of its influence on progression and offering insights into novel therapeutic strategies.

## 2. Aberrant Lactate Production in Pancreatic Cancer

The oncogenic factors lead to the activation of proto-oncogenes [35], such as kirsten rat sarcoma viral oncogene homolog (KRAS) (90%) [8,36], and the inactivation of tumor suppressor genes, such as p53 (TP53) (71%) [37,38], cyclin dependent kinase inhibitor 2A (CDKN2A) (24%), and SMAD family member 4 (SMAD4) (17%) [39], in pancreatic cells. These alterations disrupt the regulation of cell growth, which easily initiates the onset of carcinogenesis. The activation of KRAS significantly upregulates glycolysis [40,41,42]. This metabolic rewiring results in substantial lactate accumulation, with concentrations in PDAC tissues reaching 20–40 μmol/mg, a 3 to 4-fold increase over levels in adjacent normal tissues (10–20 μmol/mg) [30,43]. Consistently, the glucose transporters (GLUT) on the membrane of pancreatic cancer cells are overexpressed, facilitating rapid glucose transport into the cells. Once inside the cells, glucose is metabolized via glycolysis to produce pyruvate, which is then catalyzed by lactate dehydrogenase (LDH). While this reaction primarily regenerates NAD+ under anaerobic conditions to sustain glycolysis, in pancreatic cancer cells, it is hyperactive and produces lactate constitutively even in oxygen-rich conditions [44,45]. This conversion provides a rapid energy supply for the cells and triggers lactate accumulation in the cells [20,46].

In addition to glycolysis, pancreatic cancer cells can generate lactate via glutamine (Gln) metabolism. Specifically, pancreatic cancer cells exhibit a phenomenon called ‘Gln addiction’, driven by arginine (Arg) depletion in the microenvironment and KRAS mutations [47,48]. Consistent with this, the expression of Gln metabolism-related proteins, such as alanine-serine (Ser)-cysteine (Cys) transporter 2 (ASCT2/SLC1A5) and glutaminase 1 (GLS1), is significantly higher in pancreatic cancer cells than in normal cells. Gln is absorbed and catabolized into glutamate (Glu) and ammonia by glutaminase in the cytosol. Subsequently, Glu enters tricarboxylic acid (TCA) cycle and is further converted to α-ketoglutarate (α-KG). A series of reactions in the malate–pyruvate pathway are responsible for the conversion of α-KG to lactate [49,50]. During Gln metabolism, several factors upregulate lactate production [51,52]. Ammonia can modulate LDH activity by increasing intracellular pH [53]. Moreover, Gln metabolism is closely associated with other metabolic pathways, such as glycolysis [54] and the TCA cycle [50]. These pathways are coordinately regulated to promote lactate production. When excessive lactate accumulates in cells, it is transported to the extracellular environment via monocarboxylate transporter 1 (MCT1) and connexin-43 (CX-43) [55] to maintain the intracellular and extracellular acid–base homeostasis.

## 3. A Novel Protein Modification: Lactylation

Aberrant lactate metabolism in tumors is not limited to energy supply. Its derived epigenetic modifications also aggravate the malignant phenotype of pancreatic cancer. In 2019, Zhang et al. [56] conducted a groundbreaking study demonstrating that lactate implicates in a distinct histone modification, termed lactylation. In 2020, Galligan et al. [57] identified a novel type of lysine lactylation which is derived from a non-enzymatic acyl-transfer of lactyl-glutathione (LGSH), a glycolytic metabolite. Thus, the phenomenon of lactate covalently attaching to lysine residues of proteins is named lactylation in recent studies [58,59].

### 3.1. The Mechanism of Lactylation

**Figure 1:**
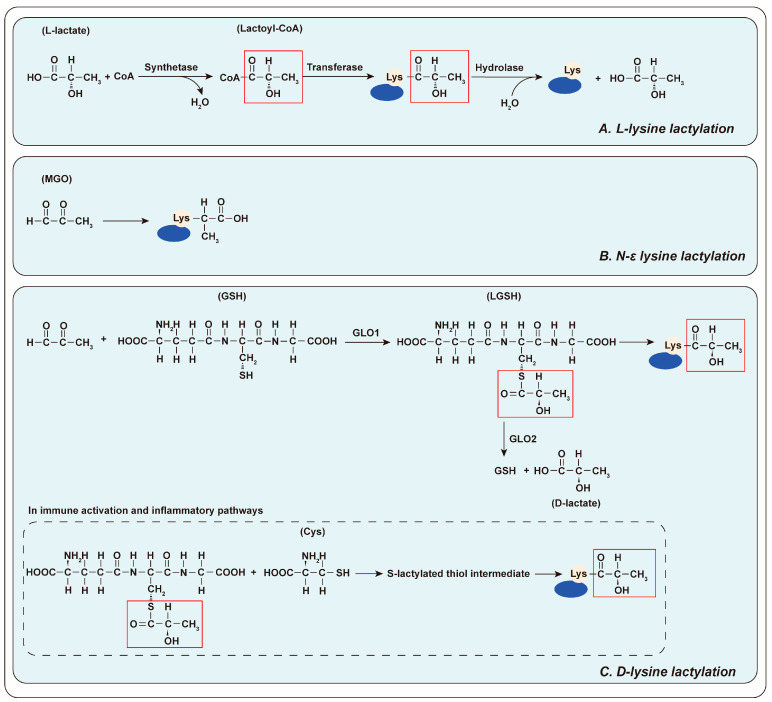
**The mechanism of lactylation**. (**A**) L- lysine lactylation: Lactate and CoA combine to form Lactyl-CoA, catalyzed by specific synthetic enzymes. Subsequently, specific transferase enzymes transfer the lactyl group to protein lysine, initiating the lactylation process. (**B**) N-ε lysine lactylation: MGO reacts directly with lysine. (**C**) D-lysine lactylation: Under the catalysis of GLO1, MGO initially reacts with GSH to form LGSH, which is followed by the transfer of the lactyl moiety to lysine, forming the D-lysine lactylation modification. LGSH also can be catabolized into GSH and D-lactate by GLO2. During innate immune activation, NF-κB signaling attenuates GLO2 expression, resulting in the accumulation of its substrate LGSH in the cytoplasm. LGSH undergoes a reversible nucleophilic substitution with adjacent Cys, forming S-lactylated thiol intermediates. These intermediates can subsequently experience an S-to-N acyltransfer reaction, transferring the lactyl group to a proximal lysine residue, completing D-lysine lactylation.

Precise mechanism of lactylation remains incompletely elucidated. Currently, it is widely accepted that lactylation is modulated through both enzymatic and non-enzymatic mechanisms. The enzymatic pathway entails the combination of lactate with coenzyme A (CoA) to form lactyl-CoA catalyzed by specific synthetases. Lactyl-CoA is then utilized for lactylation through the transfer of the lactyl moiety to lysine residues of proteins by specific transferases [60]. The enzymatic pathway is considered to mainly regulate L- lysine lactylation. L-lysine lactylation is the most prevalent form of lactylation in mammalian cells and serves as a major responder to glycolysis and the ‘Warburg effect’ [60] (Figure 1A). The number of identified lactyl-CoA synthetases is limited. Only two types, acetyl-CoA synthetase short chain family member 2 (ACSS2) [61] and GTP-specific succinyl-CoA synthetase (GTPSCS) [62], have been reported (Table 1). The lactyltransferases, a subset of lysine acetyl-transferases (KATs), can transfer multiple acyl groups rather than being restricted to a single type. Similar to KATs, the known lactyltransferases predominantly belong to three families (Table 1): E1A-binding protein p300 (p300) [43,63]/CREB-binding protein (CBP) [64], GCN5-related N-acetyltransferases family (GNAT) [61] and MOZ, Ybf2/Sas3, Sas2, and TIP60 family (MYST) [65]. Among these, general control non-depressible 5 (GCN5/KAT2A) [61], MYST histone acetyl-transferase 1 MYST1/MOF/KAT8) [66], MYST2/HBO1/KAT7 [65] and 60 kDa tat-interactive protein (TIP60/KAT5) [67] have been demonstrated to transfer lactyl groups. In contrast to these enzymes, alanyl-tRNA synthetase 1 (AARS1) [68] and AARS2 [69] function as both L-lactate receptors and lactyltransferases [58]. Surprisingly, Sun et al. [70] uncovered that histone deacetylase 6 (HDAC6) exhibits lactyltransferase activity in cells, which depends on lactate concentration and its intrinsic deacetylase activity. However, HDAC 1-3 [67,71] and SIRT1-3 [63,72,73,74,75] are known as delactylases. HDAC1-3 was found to de-lactylate both L-lysine lactylation and D-lysine lactylation [76]. These distinct enzymatic activities of HDAC6 and HDAC1-3, as well as SIRT1-3, imply a complex regulatory network in post-translational modifications (PTMs). Further research is required to precisely determine the spatial and temporal regulation of these enzymes within cells. It remains unclear under what specific physiological or pathological conditions the balance between lactyltransferase and delactylase activities is switched. In the study conducted by Sun et al. [70], HDAC6 tends to exhibit greater lactyltransferase activity under high-lactate conditions, whereas its deacetylase activity is more pronounced under low-lactate conditions. The precise molecular mechanisms underlying this regulation, as well as whether this mechanism applies to other members of the HDAC family or other enzymes, are still unknown.

Another mode of lactylation, the non-enzymatic reaction, does not necessitate the involvement of enzymes. This mode is also referred to as lactate-independent lactylation Non-enzymatic lactylation is accomplished through two principal pathways. One pathway comprises the direct reaction of methylglyoxal (MGO), a byproduct generated during glycolysis, with lysine to form carboxyethyl (N-ε lysine) lactylation (Figure 1B). The other pathway is mediated by LGSH, which is generated through the reaction of MGO with glutathione (GSH), catalyzed by glyoxalase I (GLO1). LGSH then undergoes a non-enzymatic S-to-N acyltransfer reaction, transferring the lactate moiety to lysine to form the D-lysine lactylation modification. LGSH can be catabolized into GSH and D-lactate by GLO2 [78,79,80,81] (Figure 1C). Furthermore, a study under the context of innate immune activation showed that NF-κB signaling attenuates GLO2 expression, resulting in the accumulation of its substrate LGSH in the cytoplasm. LGSH undergoes a reversible nucleophilic substitution with adjacent Cys, forming S-lactylated thiol intermediates. These intermediates can subsequently experience an S-to-N acyltransfer reaction, transferring the lactyl group to a proximal lysine residue, thereby completing D-lysine lactylation modification [78] (Figure 1C). Notably, this study was performed under the specific context of innate immune activation. And this reaction depends on the distance between the Cys and substrate lysine. Further investigation is required to explore whether alternative mechanisms of lactylation exist beyond those mentioned above.

The discovery of protein lactylation has expanded the scope of biological research on PTMs and offered valuable insights into the molecular mechanisms by which lactate influences a myriad of physiological and pathological processes, ranging from developmental anomalies [82,83] and neurodegenerative disorders [84,85] to inflammation [86,87,88] and cancer [89,90]. As a result, there has been a recent upsurge in research interest, culminating in a deeper understanding of the molecular mechanisms and regulatory functions of lactylation. Moving forward, our concentration will shift to investigating the role of lactylation in pancreatic cancer.

### 3.2. Lactylation in Pancreatic Cancer

Dong et al. [77] revealed that the expression of histone H3K18la (H3 K18 site lactylation) is significantly elevated in pancreatic cancer following lactate accumulation, which promotes the transcription of its downstream genes threonine tyrosine kinase (TTK) and BUB1 mitotic checkpoint serine/threonine kinase B (BUB1B). The activation of TTK and BUB1B elevates p300 expression. The amino acid residues in the p300 substrate binding site stably bind to lactyl-CoA, which enables p300 to catalyze the transfer of lactyl groups to histone lysine residues and achieves lactylation. Additionally, p300 can synergize with the histone chaperone protein anti-silencing function 1A (ASF1A) to regulate the histone lactylation [77]. TTK also phosphorylates LDHA, increasing lactate production. These progresses establish a positive feedback loop of glycolysis-H3K18la-TTK/BUB1B [43].

While histones can be lactylated, an increasing number of studies indicate that non-histone proteins are also subject to lactylation. In pancreatic cancer, nucleolar and spindle-associated protein 1 (NUSAP1) has been found to form a transcriptional regulatory complex with c-MYC and HIF-1α. This complex localizes to the LDHA promoter region to enhance the expression of LDHA. Lactate-induced lactylation inhibits the degradation of NUSAP1, forming a positive feedback loop of NUSAP1-LDHA-glycolysis-lactate, which promotes pancreatic cancer metastasis and negatively affects the prognosis of pancreatic cancer [27]. Huang et al. [80] confirmed that the lactylation of transcription factor EB (TFEB) at K91 impeded the interaction between TFEB and WW domain containing E3 ubiquitin protein ligase 2 (WWP2). This mechanism blocked TFEB ubiquitination and its subsequent proteasome degradation, increasing TFEB activity and augmenting the autophagy flux. Huang et al. [91] demonstrated a significant rise in the lactylation level of all-pantothenyl-lysine in patients with pancreatic adenocarcinoma. They also found that lactylation alters the properties of nucleolar and spindle-associated protein 1 (NMNAT1), which efficiently catalyzes the conversion of nicotinamide mononucleotide (NMN) to nicotinamide adenine dinucleotide (NAD+). These changes sustain the normal operation of NAD+ salvage synthesis pathway in the nucleus and inactivate p-38 mitogen-activated protein kinase (MAPK) signaling, inhibiting DNA damage inducible transcript 3 (DDIT3) transcription and ultimately promoting tumor growth. Another key player, AARS1 [68], was identified to bind lactate, catalyze the formation of lactate-AMP, and then transfer lactate to K120 and K139 in the DNA-binding domain of p53. This results in p53 lactylation, which in turn impedes the liquid–liquid phase separation (LLPS) of p53, and transcriptional activation of its target genes. Eventually, this inhibits the tumor-suppressive function of p53 [68].

Crucially, intracellular lactate in non-small cell lung cancer (NSCLC) facilitates extracellular lipolysis to produce free fatty acids by stabilizing apolipoprotein C2 (APOC2) via the lactylation of APOC2 K70. This mechanism enhances tumor metastasis and immunotherapy resistance [92]. The lactate-induced K172 lactylation of discoidin, CUB and LCCL domain containing 1 (DCBLD1) was found to stabilize DCBLD1 expression. DCBLD1 inhibits glucose-6-phosphate (G6PD) autophagic degradation and activates pentose phosphate pathway (PPP), promoting cervical cancer progression [81]. In glioblastoma, lactylated X-Ray repair cross complementing 1 (XRCC1) exhibits an increased affinity for importin α, facilitating the nuclear translocation of XRCC1 and subsequently augmenting DNA repair processes to regulate cancer cell growth [59,79]. Han et al. [93] found that ATP-binding cassette subfamily F member 1 (ABCF1) was lactylated in the nucleus, where it binds to the promoter region of lysine demethylase 3A (KDM3A). This binding upregulates KDM3A expression and promotes hepatocellular carcinoma (HCC) malignant progression through the transcriptional activation of the HIF signaling pathway. Although the underlying mechanisms remain to be fully elucidated in pancreatic cancer models, research has proved that ApoE [94], DCBLD1 [81] and XRCC1 [95] are significant factors in pancreatic cancer. Consequently, it is reasonable to hypothesize that similar non-histone lactylation mechanisms may be present in pancreatic cancer and await exploration. Consistent with this, preliminary data from our unpublished studies on the PANC-1 cell lines, utilizing label-free qualitative lactylation-modified proteomics, identified 3913 sites across 1387 proteins, comprising 43 histone and 19 non-histone proteins. However, the number of proteins exhibiting lactylation in pancreatic cancer does not reach this figure, indicating that many mechanisms remain unknown.

### 3.3. Lactate-Regulated Other Epigenetic Modification Processes

The influence of lactate extends beyond lactylation. Lactate also exerts direct or indirect effects on other epigenetic modification processes, including DNA methylation [96] and histone acetylation [96,97]. For example, lactate metabolism can directly affect the DNA methylation level by influencing the intracellular concentration of the methyl donor S-adenosylmethionine (SAM). Alternatively, lactate can be converted to pyruvate by lactate dehydrogenase (LDH), which then enters the tricarboxylic acid (TCA) cycle, generating nicotinamide adenine dinucleotide (NADH) and flavin adenine dinucleotide (FADH_2_). These molecules modify the intracellular redox state, thereby affecting DNA methyltransferases (DNMTs) activity [97].

### 3.4. Lactylation and Acetylation

An intriguing aspect is the considerable overlap between lactylation and acetylation. Both processes occur on lysine residues and share many catalytic enzymes [56,98,99]. The KATs previously mentioned can also participate in lactylation [43,63,64]. However, they are not entirely identical. Acetylation involves the transfer of an acetyl group from acetyl-CoA to lysine [100], whereas lactylation involves the attachment of a lactyl group to lysine [58,59]. The acyl donors utilized in these two processes are different. Many mysteries about the coordinated regulation of lactylation and acetylation remain. During embryo development, cells undergo rapid differentiation and proliferation. It is unclear whether the regulatory mechanisms governing the balance between lactylation and acetylation distinguish from those in normal state. The potential associations between numerous small metabolic molecules and these two modification processes have not been thoroughly investigated. Conceivably, these small molecules modulate lactylation and acetylation by influencing enzyme activities or substrate accessibility. However, this requires extensive experimental validation. Regarding future research directions, efforts should focus on developing highly sensitive detection techniques for the real-time and precise monitoring of the dynamic changes in lactylation and acetylation. This would provide a robust tool for a deeper understanding of the regulation of balance between lactylation and acetylation. Systematic studies on specific cellular physiological and pathological states, such as alterations in nerve cells during neurodegenerative diseases, could elucidate the causal relationship between the imbalance of these two PTMs and disease progression [101]. This line of research could also uncover novel targets and strategies for disease diagnosis and treatment.

## 4. Effects of Lactate Metabolism on the Microenvironment of Pancreatic Cancer

Lactate is not only an important substrate for lactylation but also plays a significant role in cellular metabolism. As research on lactate metabolism deepens, its role in the physiological processes of pancreatic cancer has increasingly come to the forefront, particularly concerning the metabolic reprogramming of tumor microenvironment [102]. This insight further paves the way for novel research avenues aimed at exploring the interplay between lactate metabolism and pancreatic cancer. In the following sections, we will elaborate on the regulatory mechanisms of lactate in pancreatic cancer cell signaling, concentrating on metabolic reprogramming, angiogenesis, and immune escape.

### 4.1. Metabolism Reprogramming

**Figure 2:**
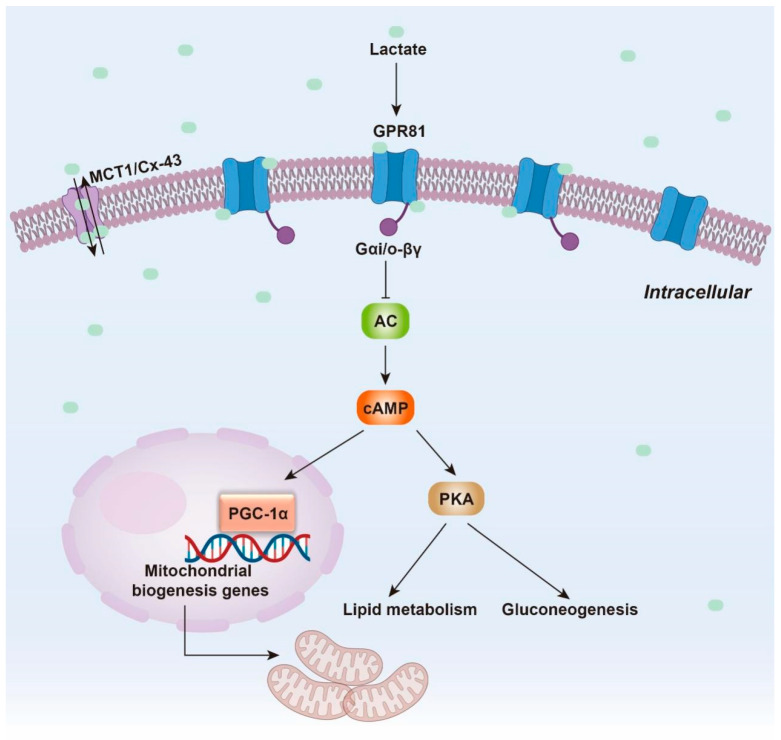
**Lactate regulates the GPR81-cAMP signaling pathway, driving metabolic reprogramming in pancreatic cancer.** GPR81, highly expressed on the membrane of pancreatic cancer cells, is activated by binding its ligand, lactate. This activation triggers its interaction with the Gαi/o and inhibits adenylyl cyclase (AC) activity via the Gαi/o subunit, reducing intracellular cAMP production. Reduced cAMP levels modulate cAMP-dependent signaling pathways, such as PKA, inhibiting the expression and activity of gluconeogenesis-related enzymes and suppresses lipolysis. In addition, cAMP regulates mitochondrial biogenesis through PGC-1α.

In pancreatic cancer cells, the expression level of G protein-coupled receptor 81 (GPR81) is significantly upregulated on the cell membrane. Lactate, a natural ligand for GPR81, binds to and activates GPR81, triggering a series of downstream MAPK signaling cascades. One of these cascades induces the inhibition of cyclic adenosine monophosphate (cAMP) production via G protein α i/o (Gα i/o). The reduction in cAMP levels diminishes the expression and activity of gluconeogenesis-related enzymes [103] and protein kinase A (PKA) [104], resulting in alterations in glucose and lipid metabolism [105,106,107]. Decreased cAMP also promotes the expression of peroxisome proliferator-activated receptor gamma coactivator 1α (PGC-1α), a key regulator of mitochondrial biogenesisPGC-1α upregulates the expression of mitochondrial DNA polymerase γ (POLG) and mitochondrial transcription factor A (TFAM), which are essential for enhancing mitochondrial quantity and function. This process supports the energy metabolism and growth of cancer cells by providing the necessary mitochondrial capacity to meet their increased metabolic demands [108,109] (Figure 2).

As pancreatic tumors develop, the metabolic profile of cancer-associated fibroblasts (CAFs) in the tumor microenvironment is significantly altered. Activated CAFs shift from an energy supply mode predominantly reliant on oxidative phosphorylation (OXPHOS) to a highly active glycolytic state, characterized by enhanced glucose uptake capacity. Glucose is metabolized into pyruvate through glycolysis, which is subsequently converted into lactate under the catalysis of LDH. Studies have shown that a portion of the lactate produced can be transported to neighboring cancer cells [110,111]. To ensure the normal metabolic activity, lactate can be transported across the cell membrane under different conditions [46,112,113]. When intracellular pH is excessively low, pancreatic cancer cells expel lactate to the extracellular environment through MCT1 and MCT4 to maintain intracellular pH stability [25,114,115]. Conversely, when intracellular energy supply is insufficient, lactate can be re-imported into the cell and converted back into pyruvate through the reverse reaction catalyzed by LDH. This pyruvate is then incompletely oxidized in the mitochondria to produce acetyl-CoA, which enters the TCA cycle to replenish energy and maintain the ATP level necessary for rapid cancer cells proliferation [31,116]. Collectively, lactate is not a waste product but a versatile metabolic substrate that can be utilized for oxidation, biosynthesis, and signaling, thereby orchestrating the metabolic reprogramming of pancreatic cancer.

### 4.2. Angiogenesis

The rapid growth of cancer cells subjects pancreatic cancer to a hypoxic and nutrient-deprived environment. To survive and perpetuate their growth under such adverse conditions, cancer cells initiate a series of adaptive mechanisms, with angiogenesis being a pivotal strategy. Angiogenesis is defined as the formation of new blood vessels from pre-existing ones, driven by the proliferation, migration, and differentiation of endothelial cells [117,118,119]. This intricate process occurs in various physiological and pathological contexts, including embryonic development, tissue repair, and tumor progression [120]. Under normal physiological conditions, angiogenesis is tightly regulated, maintaining a relatively balanced state [121]. Nonetheless, this equilibrium is disrupted in pathological conditions such as pancreatic cancer, where tumor cells exhibit exponential proliferation, highly active metabolism, and an increased demand for essential nutrients, including oxygen, glucose, amino acids, and fatty acids [122,123]. This hostile environment prompts tumor cells and tumor-associated macrophages to secrete a range of pro-angiogenic factors [11,24]. These newly formed blood vessels continuously transport essential nutrients to support robust tumor cell proliferation and division, activate multiple intracellular signaling pathways, and promote proliferation, differentiation and migration. Beyond nutrient supply, the neovasculature can also deliver a variety of growth factors that further enhance tumor growth [120,124].

**Figure 3:**
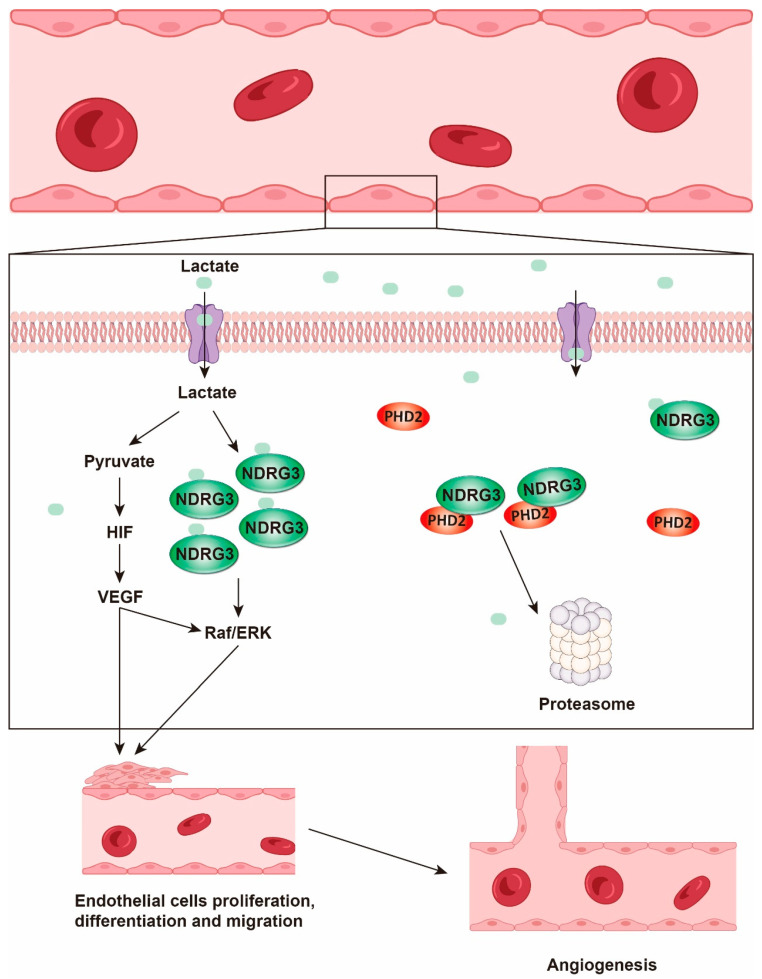
**Angiogenesis**. Upon entering endothelial cells, lactate is oxidized to produce pyruvate, which stabilizes HIF-1α and upregulates the key angiogenic factor VEGF. Furthermore, lactate directly binds to NDRG3, preventing its degradation and promoting its accumulation. This activates the Raf/ERK pathway, increasing the expression of angiogenesis-related genes and accelerating endothelial cell proliferation, differentiation and migration, ultimately facilitating new blood vessel formation.

Lactate transported into endothelial cells is oxidized to pyruvate by LDH, stabilizing HIF-1α and subsequently upregulating vascular endothelial growth factor (VEGF), a master regulator of angiogenesis [28,125]. VEGF stimulates the proliferation and migration of endothelial cells [126] and induces pancreatic cancer cells to shift from mitochondrial OXPHOS metabolism to glycolysis, generating more lactate and creating a positive feedback loop [127,128]. Furthermore, lactate can directly bind to N-MYC downstream-regulated gene 3 (NDRG3), preventing its degradation by prolyl hydroxylases 2 (PHD2). This binding leads to the intracellular accumulation of NDRG3 and activation of the Raf/extracellular signal-regulated kinase (Raf/ERK) pathway, which promotes the expression of various angiogenesis-related genes and accelerates tumor neovascularization, providing favorable conditions for rapid tumor growth and metastasis [129] (Figure 3).

### 4.3. Immune Evasion

To persist and successfully metastasize to distant tissues and organs, tumor cells must evade the immune system [130]. In pancreatic cancer, immune evasion presents a significant clinical challenge [131,132]. Tumor cells evade recognition and attack by the immune system through multiple mechanisms. One key strategy involves the expression of specific protein molecules on their surface [132,133]. These molecules include programmed death-ligand 1 (PD-L1) [134], cytotoxic T lymphocyte-associated protein 4 (CTLA-4) [135], ecto-5′-nucleotidase (CD73) [136], and epidermal growth factor receptor (EGFR) [137]. These molecules bind to receptors on immune cells, suppressing their activity and impeding the effective elimination of tumor cells [137]. In recent reports, lactate-activated GPR81 was found to upregulate PD-L1 expression, driving tumor cell immune evasion [138,139]. Additionally, lactate within the tumor is recognized by AARS1, which lactylates two key amino acids in the DNA-binding domain of oncoprotein p53. This lactylation inactivates p53 and promotes cancer progression [68].

The stromal cells, immune suppressive cells, and ECM surrounding the tumor also create an environment conducive to tumor survival and escape [140]. Pancreatic stellate cells (PSCs) secrete large quantities of ECM components, such as collagen and fibronectin, forming a dense stromal barrier that prevents the penetration of drugs and immune cells [9,141]. CAFs secrete various growth factors and cytokines, such as interleukin-6 (IL-6) and IL-8, which remodel the microenvironment and promote tumor growth, invasion, and metastasis [10,142]. Myeloid-derived suppressor cells (MDSCs) secrete immune suppressive factors like arginase and nitric oxide (NO), which inhibit the function of T cells and natural killer (NK) cells [143]. Regulatory T cells (Tregs) alleviate effector T cell activity, thereby reducing the immune system’ s attack on tumor cells [144].

Lactate binding to GPR81 on macrophages drives their polarization from the pro-inflammatory M1 phenotype to the immunosuppressive M2 phenotype. Simultaneously, lactate-induced histone lactylation further upregulates the expression of arginase 1 (Arg1) [145], a key marker of M2-type macrophages (tumor-associated macrophages, TAMs). The increased expression of Arg1 contributes to the conversion of TAMs from the M1 to the M2 phenotype [146,147]. This conversion is achieved through the secretion of transforming growth factor-beta (TGF-β) [148] and IL-10 [149], as well as the regulation of angiogenesis, lymphangiogenesis, and restriction of CD8^+^ T-cell infiltration via various mechanisms [89,150].

Tumor cells experience profound metabolic stress, which drives high rates of glycolytic flux and lactate production [151,152]. The resulting accumulation of lactate within the tumor microenvironment impedes the lactate export from tumor-infiltrating T cells, leading to dysfunctional T cell metabolism and reduced anti-tumor responsiveness [46]. This phenomenon helps explain the differential effectiveness of T-cell-based therapies in hematological malignancies compared to solid tumors. The acidic and hypoxic conditions the tumor microenvironment of solid tumors exert significant immunosuppressive effects, posing a challenge to T cell functionality [153,154].

**Figure 4:**
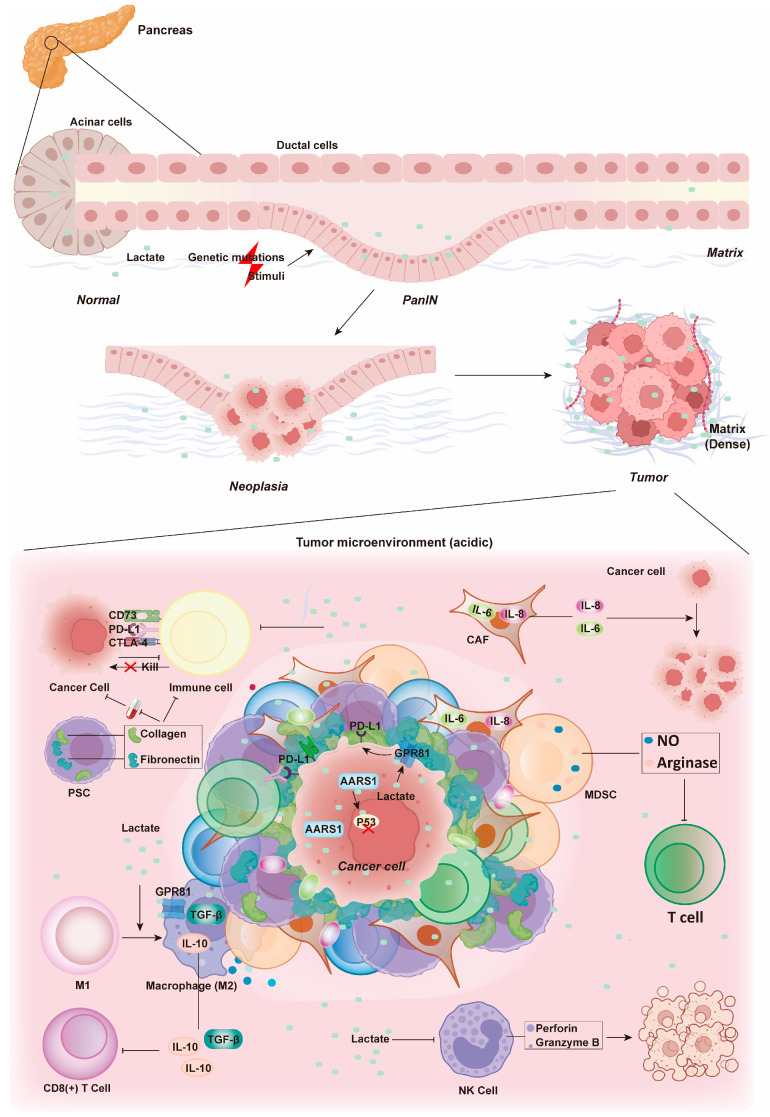
**The evolution of pancreatic cancer and immune evasion mechanisms.** Pancreatic cells are susceptible to pathological lesions and potentially progress to neoplasia and tumorigenesis due to internal genetic mutations or external stimuli. During this progression, tumor cells undergo metabolic reprogramming. This leads to acidification of the tumor microenvironment. Notably, lactate production inhibits immune cells in the microenvironment, contributing to tumor cell immune evasion.

A study using a murine pancreatic cancer model revealed that lactate treatment decreased the production of perforin and granzyme B by NK cells [155]. These substances are vital for the cytotoxic and cytolytic function of NK cells, which eliminate cancer cells by releasing lytic granules. The reduction in perforin and granzyme B directly compromises NK cell function [156], promoting pancreatic cancer progression [157]. Lactate also significantly impacts dendritic cells (DCs), which capture, process and present tumor-associated antigens to T cells, thereby activating specific T-cell immune responses. DCs serve as an important bridge between innate and adaptive immunity [158]. Lactate suppresses the differentiation of monocytes into DCs [159], rendering these cells tolerogenic and enhancing the production of the immunosuppressive cytokine IL-10 [158]. Moreover, lactate potently suppresses the antigen-presenting function of mature DCs through the GPR81-mediated signaling pathway (as described in Section 4.1) (Figure 2). The subsequent suppression of the cAMP-PKA axis promotes the ubiquitin-proteasome-dependent degradation of internalized antigens and downregulates the expression and assembly of major histocompatibility complex class I (MHC-I) molecules [158]. MHC-I presents endogenous antigenic peptides to CD8^+^ T lymphocytes, enabling these T cells to recognize and eliminate abnormal cells, such as virus-infected cells or tumor cells [160]. Therefore, lactate-induced antigen degradation impairs the immune system’s ability to eradicate abnormal cells during tumorigenesis (Figure 4). It is evident that the immune evasion of tumor cells mediated by lactate is crucial for the persistence and metastasis of pancreatic cancer.

## 5. Diagnosis of Pancreatic Cancer Based on Lactate Metabolism

Lactate metabolism plays a vital role in the complex regulatory mechanisms underlying pancreatic cancer. Building on foundational research into lactate metabolism in the context of pancreatic cancer, it is highly plausible that lactate metabolism-related indicators may emerge as prospective biomarkers for early diagnosis or as effective therapeutic targets in future clinical applications. In recent years, research on lactate metabolism in pancreatic cancer has gained significant traction. The following section will discuss the prospects of targeting lactate metabolism for the diagnosis and treatment of pancreatic cancer.

Early-stage pancreatic cancer typically presents with no overt symptoms, and by the time symptoms become apparent, the disease is often already in its intermediate or advanced stages. Traditional imaging modalities, such as ultrasound and computed tomography (CT), are unable to detect early-stage abnormalities promptly. Although the tumor marker carbohydrate antigen 19-9 (CA19-9) offers high sensitivity [161], its specificity is relatively low. Leveraging the metabolic characteristics of pancreatic cancer cells, such as lactate metabolism discussed in this paper, as a diagnostic indicator for pancreatic cancer could enable the detection of abnormalities during the asymptomatic phase, providing the potential for early intervention.

Research into targeting lactate metabolism for pancreatic cancer diagnosis has shown promising results. Serum marker monitoring has identified lactate as a differential metabolite in pancreatic cancer patients across different stages [159,162], implying that lactate may exhibit high sensitivity and specificity for pancreatic cancer diagnosis and could serve as a potential diagnostic biomarker. For instance, under physiological conditions, serum lactate concentration is tightly maintained within a range of 1.5–3.0 mM. In stark contrast, the tumour microenvironment of cancer exhibits profound lactate accumulation, with concentrations reaching 10–50 mM [163]. Studies combining the monitoring of lactate, pyruvate, and circulating nucleosomes have demonstrated that the differential expression levels of these three markers in the serum of pancreatic cancer patients compared to healthy individuals are statistically significant. This finding suggests a potential direction for identifying new biomarkers for pancreatic cancer. Magnetic resonance spectroscopy (MRS) enables the real-time detection of metabolite levels in living tissues and may aid in the diagnosis of pancreatic cancer by detecting lactate [164]. Hyperpolarized 13C MRS technology is an emerging imaging modality that allows for the real-time acquisition of dynamic metabolic data on the hyperpolarized pyruvate-lactate transition in vivo, following the injection of hyperpolarized [1-13C] pyruvate probes [164,165]. The accumulation of lactate is positively correlated with the pH of the tumor microenvironment, leading to the design of molecular imaging probes responsive to acidic conditions. These probes can image the weakly acidic tumor microenvironment of pancreatic cancer, indirectly reflecting the lactate content of the tumor microenvironment and aiding in diagnosis.

While these probes hold certain application value, they also have limitations. Some probes may be metabolized and degraded in the body or induce immune reactions, potentially affecting imaging quality. Additionally, the tumor microenvironment of pancreatic cancer is characterized by dense mesenchymal tissue, which hinders the uniform penetration of probes into tissues for effective detection. Coupled with inherent limitations in resolution and sensitivity, there is an increased risk of misdiagnosis and missed diagnosis. Consequently, the optimization of imaging technology remains an area that warrants further exploration.

## 6. Targeting Lactate Metabolism in Pancreatic Cancer Therapy

### 6.1. Inhibiting Lactylation

Lactate metabolism, beyond its diagnostic potential, has emerged as a promising therapeutic avenue for pancreatic cancer. This metabolic pathway is intricately regulated through proteins lactylation, primarily via the targeting of lactate transferase AARS or the delactylases HDAC and SIRT. However, research on lactate transferase remains limited. For instance, β-alanine has been shown to competitively bind to AARS1, inhibiting its lactylation activity [32]. Meanwhile, HDACs, members of the deacetylase family, have been extensively studied, with inhibitors such as sodium butyrate and trichostatin A (TSA) capable of suppressing HDAC activity. This suppression elevates protein lactylation levels, significantly influencing pancreatic cancer progression. Nevertheless, achieving therapeutic success through the modulation of lactate metabolism via lactylation presents substantial challenges. HDACs function as both delactylases and deacetylases, and their inhibition can affect multiple metabolic processes. Therefore, the meticulous evaluation of potential off-target effects is essential to minimize drug side effects when employing HDAC inhibitors as therapeutic strategies [32]. Overall, research on lactylation is still far from clinical application. Future efforts should focus on identifying key molecules involved in lactylation to elucidate its mechanisms at different stages of pancreatic cancer development, metastasis, and recurrence. In addition, overcoming targeting and specificity issues in inhibitor development is crucial. Rational drug design and optimization can help minimize interference with normal metabolism, thereby precisely targeting cancer-associated abnormalities in lactate metabolism (Table 2).

### 6.2. Inhibiting Lactate Synthesis: Targeting LDH

Existing studies have predominantly concentrated on inhibiting LDH, a key enzyme in lactate synthesis, to reduce lactate production and mitigate its tumor-promoting effects. Several inhibitors have been identified, including oxamate, which competitively binds to the active site of LDHA, displacing pyruvate and significantly reducing lactate production in pancreatic cancer cells [151,171]. This inhibition suppresses tumor cell proliferation, migration, and invasion, both in vitro and in vivo [172]. In animal models, oxamate treatment has been associated with extended survival in hormonally treated animals [173]. Another LDHA inhibitor, FX11, has demonstrated tumor growth suppression in vivo and in vitro experiments [151]. Moreover, combining FX11 with the M2-type pyruvate kinase inhibitor TEPP-46 enhances tumor growth inhibition [174]. The novel pan-LDH inhibitor galloflavin uniquely inhibits both LDHA and LDHB by binding to the free enzyme without competing with substrates or cofactors. At micromolar concentrations, galloflavin blocks aerobic glycolysis, induces apoptosis, and promotes cellular death without interfering with cellular respiration [175] (Table 2).

### 6.3. Targeting Lactate Transport: The Role of MCT

As mentioned above, MCTs are indispensable for lactate shuttling in and out of cells and have been closely linked to prognosis and lymph node metastasis in PDAC [176,177]. MCT1 small-molecule inhibitors, such as AZD3965 and AR-C155858, have undergone relevant clinical trials [116,166,178,179]. AZD3965, an orally available MCT1 inhibitor, has demonstrated tumor growth inhibition in preclinical studies of small cell lung cancer [180] and shown preclinical safety in breast cancer treatment [167]. AR-C155858 inhibits lactate uptake and cell proliferation in 4T1 breast cancer cells and enhances therapeutic efficacy when combined with chimeric antigen receptor T-cell (CAR-T) therapy against B-cell malignancies [166]. Syrosingopine, a dual inhibitor of MCT1 and MCT4, blocks lactate and hydrogen ion (H^+^) efflux, raising intracellular lactate levels and reducing glycolysis [181]. It exhibits significant antitumor activity in NSCLC models [182] and potential efficacy in breast and thyroid cancers [182,183]. Notably, combining syrosingopine with metformin significantly enhances the antitumor activity. Metformin, which inhibits mitochondrial NADH dehydrogenase, further obstructs NAD^+^ regeneration, leading to glycolysis blockade. This combination therapy has revealed synthetic lethality in several cancer types [181,184] (Table 2).

### 6.4. Targeting Other Metabolic Pathways: An Alternative Strategy

Beyond direct targeting of LDH or MCTs, another viable strategy involves cascading interference with lactate transport and production via metabolic signaling pathways. In glycolysis, glucose antimetabolite 2-deoxyglucose (2-DG) competitively binds to hexokinase (HK), inhibiting its activity and blocking the glycolysis pathway [185]. Inhibitors of phosphofructokinase (PFK), such as penfluridol [186], quercetin [187], PFK15 [188] and PFK158 [169], regulate glycolysis flux by inhibiting PFK. Notably, PFK158 has been reported to synergize with cisplatin (CBPt) and paclitaxel (PTX) to enhance chemotherapeutic effects in drug-resistant cell lines [169]. TEPP-46 activates pyruvate kinase M2 (PKM2), reducing pyruvate-to-lactate conversion [189]. The small-molecule GLUT1 inhibitor WZB117 downregulates glucose transporter proteins and intracellular ATP, thereby blocking glycolysis [168]. Metformin targets HK2 to block the glycolysis [190]. In lipid metabolism, dual inhibitors of fatty acid-binding protein 4 (FABP4) and FABP5 significantly ameliorate lipid metabolism disorders, reduce fatty acid oxidation and utilization, and indirectly affect lactate production [191]. In amino acid metabolism, DRP-104 reduces glutamine-to-lactate conversion by inhibiting the glutamine metabolic pathway [170]. Although these metabolic inhibitors do not modulate lactate metabolism through a single step, biological systems regulation is a complex interconnected network. By interfering with various metabolic pathways, these strategies ultimately achieve the modulation of lactate metabolism, highlighting the potential for multi-faceted therapeutic approaches in pancreatic cancer treatment (Table 2).

## 7. Conclusions and Discussion

**Figure 5:**
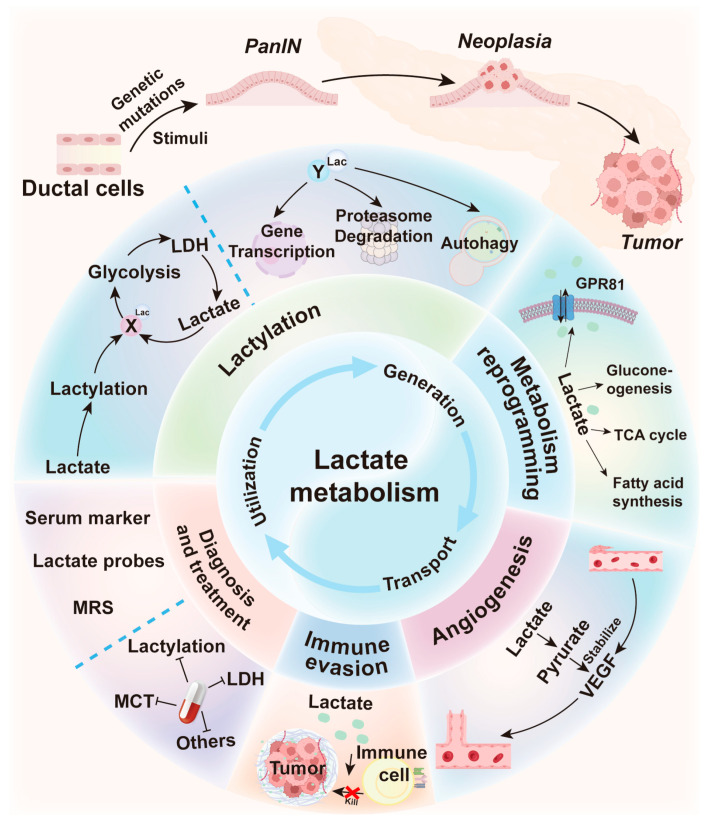
**Schematic Overview of Lactate-Centric Regulation in Pancreatic Ductal Adenocarcinoma.** During the malignant progression of pancreatic cancer, large quantities of lactate are produced and exported across the cell membrane via MCTs, serving as a substrate for gluconeogenesis, TCA cycle and fatty acid synthesis. Lactate also participates in the regulation of downstream signaling pathways through the GPR81. Furthermore, lactate accumulation in the tumor microenvironment influences angiogenesis, reprograms cellular metabolism, promotes immune escape, and consequently remodels the tumor ecosystem. Finally, we highlight the use of lactate as a serum biomarker detectable by MRS or molecular probes, as well as therapeutic strategies targeting MCTs or LDH activity.

Lactate metabolism plays a crucial role in orchestrating the process of pancreatic cancer through a multifaceted and intricate regulatory network. Lactate acts as a precursor for protein lactylation, which modifies protein structures and functions. This progress regulates downstream signaling pathways, thereby promoting tumor cell proliferation, invasion and metastasis. Additionally, lactate serves as a key substrate for various biosynthetic pathways, including lactylation, gluconeogenesis, TCA cycle and fatty acid synthesis, and functions as an agonist for the G-protein coupled receptor GPR81 to modulate cAMP/PKA signaling (Figure 5). This metabolic adaptation maintains the equilibrium of tumor cells, allowing them to thrive in nutrient-limited conditions. Lactate accumulation acidifies the tumor microenvironment, and facilitates tumor cell invasion and metastasis by activating specific signaling pathways and suppressing immune cell functions. Lactate can also remodel the tumor microenvironment by regulating the functions of CAFs and macrophages, creating a favorable environment for tumor growth and metastasis.

The critical role of lactate metabolism in pancreatic cancer development has been extensively studied, providing a theoretical foundation for developing therapeutic strategies targeting this metabolic process. Preclinical studies show that lactate metabolism inhibitors exhibit promising anticancer activity, offering a new therapeutic avenue for pancreatic cancer management. However, the clinical application of these inhibitors faces several challenges, including inadequate target specificity, which may disrupt normal energy metabolism and heighten the risk of adverse reactions. The complex signaling network of lactate metabolism in pancreatic cancer also makes it difficult for single inhibitors to comprehensively block this progress, potentially leading to drug resistance. Furthermore, the intricate metabolic environment in pancreatic cancer and the depletion of lactate metabolites through alternative pathways may reduce the efficacy of these inhibitors.

Despite these challenges, there is significant potential for optimizing lactate metabolism inhibitors. Enhancing their structural specificity can improve targeting and reduce adverse effects on normal cells. Multi-target, multi-drug combination therapies have emerged as a promising approach. By leveraging the synergistic effects of various drug mechanisms, they can effectively counteract tumor cell compensatory mechanisms. Innovative drug delivery systems offer new possibilities. Zhang et al. [192] developed lactate-responsive enzyme-assisted Janus mesoporous silica nanoparticles. These nanoparticles incorporate lactate oxidase, which specifically recognizes and decomposes lactate into pyruvate and hydrogen peroxide, triggering drug release and improving targeting and therapeutic efficacy [192]. This system demonstrates promising efficacy in preclinical models when combined with other therapies, highlighting its potential to provide novel insights for targeting lactate metabolism in pancreatic cancer treatment.

Advances in histological techniques and big data analytics allow systematic mapping of intracellular signaling pathways. These advancements not only deepen our understanding of the regulatory networks governing lactate metabolism in pancreatic cancer and their dynamic interplay with other metabolic pathways, but also facilitate the integration of single-cell metabolomics with spatial genomics. AI-driven platforms such as DeepSeek-V3 and ChatGPT 5.0 can further decode the spatiotemporal heterogeneity of lactate metabolism and identify potential therapeutic targets. Sustained investigations of lactate metabolism in pancreatic cancer can facilitate the early detection and continuous surveillance of the disease, which paves the way for innovative therapeutic avenues and effective management strategies.

In summary, this review provides an analysis of contemporary therapeutic strategies targeting lactate metabolism in pancreatic cancer (Figure 5). It elucidates foundational biological mechanisms, evaluates diverse treatment modalities, and highlights challenges and future research directions.

## Figures and Tables

**Table 1:** The enzyme for lactylation process.

Function	Name	Reference
Lactyl-CoA Synthetase	ACSS2	[61]
	GTPSCS	[62]
Lactyltransferase	P300	[63,77]
	CBP	[64]
	GCN5/KAT2A	[61]
	MYST1/MOF/KAT8	[66]
	MYST2/HBO1/KAT7	[65]
	TIP60/KAT5	[67]
	AARS1	[32,68]
	AARS2	[69]
	HDAC6	[70]
Delactylase	HDAC 1-3	[67,71,76]
	SIRT 1-3	[63,73]

**Table 2:** Summary of therapeutic strategies targeting lactate metabolism in pancreatic cancer.

Therapeutic Approach	Target	Representative Inhibitors	Mechanism	Advantages	Challenges
Inhibit lactate production	LDHA	Oxamate [163], FX11 [146]	Inhibits LDHA, reducing pyruvate-to-lactate conversion.	Targets the core of glycolytic flux, multiple inhibitors available.	Off-target effects, potential compensatory mechanisms by other LDH isoforms.
Inhibit lactate transport	MCT1/4	AZD3965 [111], Syrosingopine [166]	Blocks lactate efflux, causing intracellular acidification and glycolysis inhibition.	Target lactate export in both cancer and stromal cells, some inhibitors are in clinical trials.	MCT4 affinity issues for some inhibitors, potential hematological toxicity.
Target lactylation	AARS1 (Lactyl-transferase),	β-alanine (potential) [32]	Inhibits lactyl-transferase activity, reducing protein lactylation.	Novel epigenetic targeting, potential for high specificity.	Early stage of research, lack of potent and specific clinical inhibitors.
	HDACs (De-lactylases)	Sodium butyrate [32], TSA [32]	Inhibits de-lactylase activity, potentially hyper-stabilizing lactylated proteins.	Uses existing HDAC inhibitor, dual function (deacetylase/delactylase).	Lack of specificity, complex and unpredictable outcomes.
Target upstream pathways	Glycolysis	2-DG [167] WZB117 [168] PFK158 [169]	Inhibits key glycolytic enzymes (HK, GLUT1, PFKFB3), reducing lactate source.	Broad impact on cancer metabolism, some compounds in clinical trials.	Affect normal tissues with high glycolytic demand, compensatory metabolism.
	Glutaminolysis	DRP-104 [170]	Inhibits glutamine metabolism, reducing a key substrate for lactate production.	Targets “glutamine addiction” of PDAC, impacts multiple metabolic pathways.	Systemic glutamine depletion has side effects.

## Data Availability

No new data were created or analyzed in this study.
